# Metal-Free Aerobic C–N Bond Formation of Styrene and Arylamines via Photoactivated Electron Donor–Acceptor Complexation

**DOI:** 10.3390/molecules28010356

**Published:** 2023-01-01

**Authors:** Duona Fan, Ahmed Sabri, Hiroaki Sasai, Shinobu Takizawa

**Affiliations:** SANKEN, Osaka University, Mihogaoka, Ibaraki-shi 567-0047, Osaka, Japan

**Keywords:** hydroamination, photocatalyst-free, UV light, C–N functionalization, photoactivation

## Abstract

This study processes a facile and green approach for the Markovnikov-selective hydroamination of styrene with naphthylamine through irradiation with UV LED light (365 nm) via an electron donor–acceptor complexation between naphthylamines and oxygen in situ. This protocol showcases the synthetic potential for aerobic C–N bond formation without using a metal catalyst and photosensitizer. Three naphthylamines were examined and afforded desired C–N bond formation product in moderate yield.

## 1. Introduction

Carbon–nitrogen (C–N) bond formation is crucial and has been intensively studied for amine synthesis, comprising important chemical building blocks such as illuminated materials and organocatalysts [1,2,3,4]. Hydroamination [5] and transition metal-mediated cross-coupling of amines and aryl halides [6,7] are the most commonly applied strategies for C–N bond-forming reactions. In particular, hydroamination, which can directly connect amines and alkenes with high atom economy and accessibility, is a powerful synthetic approach for C–N bond formation. However, these methods require transition metal catalysts such as palladium, copper, and iron complexes [8,9,10,11,12]. Owing to the recent rapid progress in green chemistry, more environmentally benign and facile C–N bond formation methods have emerged, such as radical-based photocatalysis [13,14]. However, most photocatalytic hydroaminations that employ high-oxidative/reductive potential photosensitizers still depend on transition metal catalysis. In addition, no control over the generation of nitrogen-centered radicals can result in various unexpected side reactions [15]. Therefore, more effective and environmentally friendly C–N bond formation methods, especially those that involve metal-free and photosensitizer-free transformations, are in high demand [16]. In 2011, Hoffmann et al. found that irradiation with UV light promoted the catalyst-free bond formation of electron-deficient alkenes, but resulted in an excess amount of naphthylamine derivatives (15 equiv.) as coupling partners [17].

**Figure 1:**
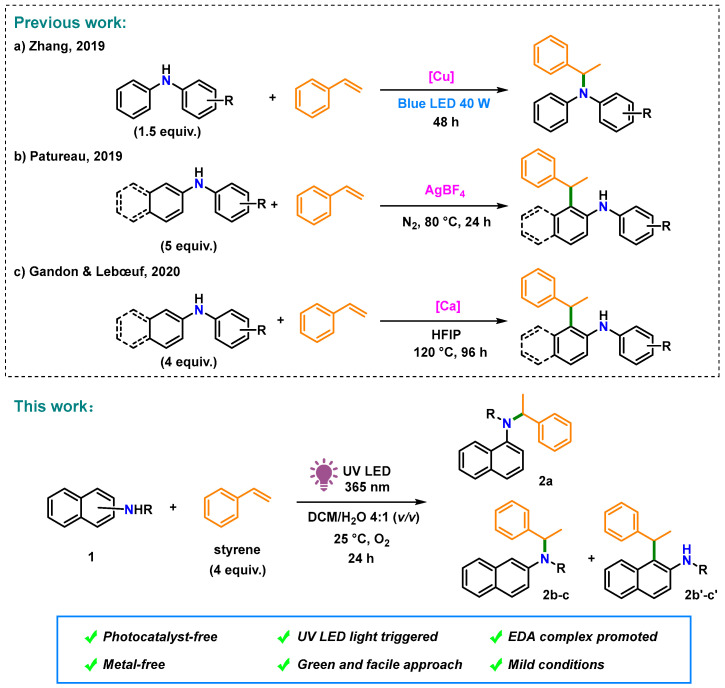
Photoinduced metal- and photocatalyst-free aerobic C–N bond formation of styrene and naphthylamine derivatives. (a) Zhang, (2019) [30]; (b) Patureau, 2019 [31]; (c) Gandon & Lebœuf, 2020 [32].

The discovery of electron donor–acceptor (EDA) complexes that facilitate photocatalyst-free transformation [18,19] has resulted in the evolution of a wide range of EDA complex-based synthetic C–N bond formation methods. Recently, an EDA complex-initiated annulation reaction of electron-deficient alkenes and alkyl anilines (electron donors) under visible-light irradiation was developed by Sundén et al. [20,21]. However, the hydroamination of arylamines with styrene is difficult because both substrates are electron donors with relatively low oxidation potentials [22]. To date, the C–N bond formation between arylamines and styrene has been accomplished mainly via reactions mediated by late-transition metals [23,24,25,26] or strong Brønsted acids [27,28,29]. Recently, naphthylamines were also included in the hydroamination reactions. In 2019, Zhang et al. reported visible-light-triggered hydroamination of styrene with arylamines using a copper catalyst and a strong base, which provides facile access to amines with Markovnikov regioselectivity (Figure 1a) [30]. Thereafter, ortho-alkylation of arylamines with styrene was performed by Patureau [31] in 2019 and Gandon in 2020 [32] using AgBF_4_ and the strong Lewis acid Ca(NTf_2_)_2_, respectively (Figure 1b,c).

For establishing metal and photosensitizer-free transformations of electron-rich substrates, herein, we report the photocatalytic Markovnikov-selective hydroamination of naphthylamines and styrene via the EDA complexation of naphthylamines and oxygen in situ (Figure 1, This work). This photochemical strategy is eco-friendly, green, and simple to perform, making it a suitable alternative to C–N bond formation under irradiation with 365 nm UV LED light in an O_2_ atmosphere.

## 2. Results and Discussions

To investigate the feasibility of the targeted photocatalyst-free reaction triggered by LED light, 1-naphthylamine (**1a**), and 2-naphthylamine (**1b**) with styrene were employed (Table 1). Remarkably, when **1a** was used in the photocatalytic reaction, *N*-(1-phenylethyl)-2-naphthylamine (**2a**, CCDC 2221784) was formed as the major product. In the case of substrate **1b**, desired product **2b** and 1-alkylated product **2b’** were obtained. The molar ratio of the starting materials was an important factor affecting the yield of desired product **2** (entries 1–3, 5, 12–14); the reaction in a 1:4 ratio of **1a** and styrene gave a 61% yield of **2a** (entry 5). Among the screened wavelengths from UV to visible light (entries 4–6), the 365 nm LED light was the most suitable for promoting the reaction (entry 5). The solvent system was also a crucial factor that significantly affected the yield of **2** (entries 7–11). After thoroughly evaluating the solvent system (also see Supporting Information), we found that when a binary solvent dichloromethane (DCM) and H_2_O was used in a ratio of 4:1 as a mixed solvent system, better catalytic performance was observed for both substrates **1a** and **1b** (entries 5 and 14), respectively. During our screening of reaction conditions, the addition of Cs_2_CO_3_ as an inorganic base could slightly improve the total yield of **2b** and **2b’** (entries 15–17) [33]. When photocatalysts were used, no reaction occurred (entries 18–21). Finally, *N*-methyl-1-naphthylamine **1c** was found to be the appropriate substrate. Under the optimized conditions, 30% yield of **2c** (C–N product) and 22% yield of **2c’** (C–C product) were obtained, as shown in entry 22.

**Figure 2:**
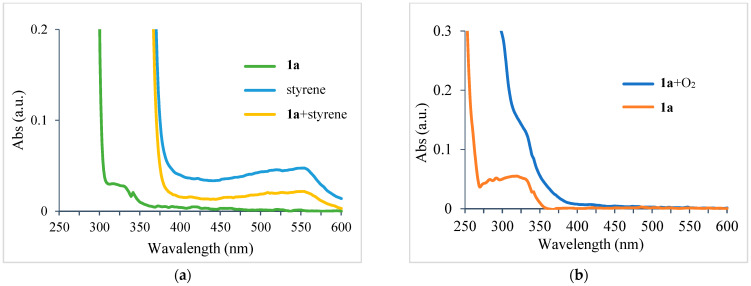
(**a**) UV–vis absorbance spectra of **1a**, styrene, and mixture of **1a** and styrene in DCM; (**b**) UV–vis absorbance spectra of **1a** and mixture of **1a** and O_2_ in DCM.

**Figure 3:**
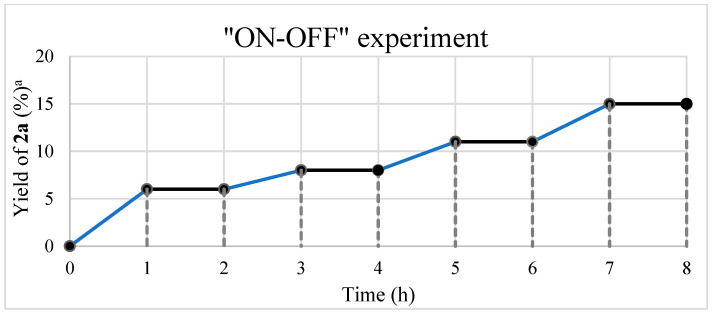
Result of the ON-OFF experiment using **1a** and styrene. Reaction conditions: **1a** (28.6 mg, 0.2 mmol) and styrene (0.09 mL, 0.8 mmol) in DCM/H_2_O (2.0 mL, 4/1) under 365 nm UV irradiation and O_2_ atmosphere.

**Figure 4:**
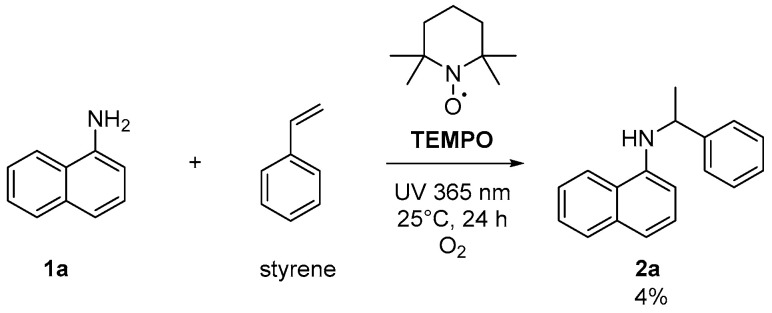
Reaction in the presence of TEMPO.

To elucidate the reaction mechanism of the proposed UV-light-promoted process, several control experiments were conducted, as shown in Figure 2 and Figure 3. Although the UV–vis spectroscopic measurements of **1a**, styrene, and a mixture of **1a** and styrene in DCM did not show any peak shifting (Figure 2a), the UV–vis spectroscopic analysis of a mixture of **1a** and O_2_ in DCM showed an increase in the bathochromic displacement and absorption (Figure 2b), which probably supported the formation of an electron donor–acceptor EDA complex between **1a** and O_2_. After mixing oxygen with naphthylamine in DCM, a color change from colorless to orange was immediately observed, which might also support EDA complex generation (also see Supplementary Materials). Next, an “ON-OFF” UV light irradiation (365 nm) experiment was conducted (Figure 3). Under light irradiation, the mixture resulted in a reaction that formed the corresponding product **2a**. However, the reaction was completely suppressed under no light irradiation. Finally, continuous 365 nm UV light irradiation resulted in the consumption of **1a** to give **2a** with 61% yield (see Supplementary Materials for details). In the presence of TEMPO (4 equiv.) as a radical scavenger, the reaction of **1a** with styrene was avoided even under the optimized conditions (Figure 4).

**Figure 5:**
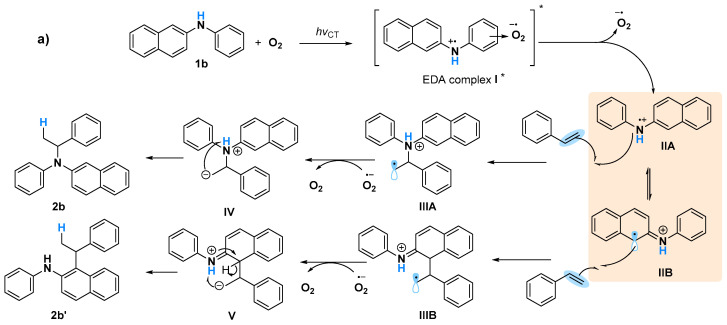

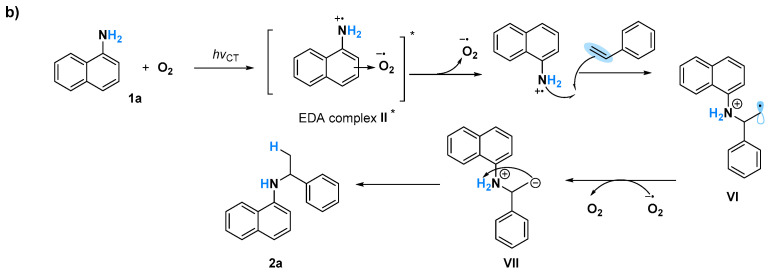
Proposed mechanism for photoinduced amination reactions using (**a**) **1b**, and (**b**) **1a**.

A Job plot study to determine the correlation of generating the EDA complex in situ with various ratios of O_2_ is challenging because of the difficulty in measuring the exact amount of O_2_ against naphthylamine, although all of these obtained results will probably be in agreement with the proposed mechanism involving the formation of naphthylamine radicals (Figure 5). Triggering of **1b** and O_2_ via UV light induced the formation of EDA complex I*. The generated **II** species might be in equilibrium with **IIA** and **IIB** via electron transfer [34], after the bond formation of styrene with **IIA** and **IIB** led to the corresponding C–N formation product **2b** and C–C formation product **2b’**, respectively [15] (Figure 5a). When **1a** was used as the substrate, only C–N formation occurred, probably owing to the sole generation of N cation radicals (Figure 5b). Although the intramolecular hydroamination to give Markovnikov products via amine radical cation species was reported [15], a naphthylamine anion radical generated by a reaction of oxygen anion radical with **1** might be another possible pathway for this C–N bond formation. Currently, the exploration of mechanism is ongoing in our laboratory.

## 3. Materials and Methods

### 3.1. Materials

1-naphthylamine (**1a**), *N*-phenyl-2-napthylamine (**1b**), and styrene were purchased from Tokyo Chemical Industries (TCI). All commercially available organic and inorganic compounds were used directly without further purification.

### 3.2. Methods

#### 3.2.1. Spectroscopy and Spectrometry

^1^H- and ^13^C-NMR spectra were recorded at 25 °C using a JEOL JMN ECS400 FT NMR instrument (^1^H-NMR 400 MHz; ^13^C-NMR 100 MHz). The ^1^H-NMR spectra are reported as follows: chemical shift in ppm downfield of tetramethylsilane and referenced to a residual solvent peak (CHCl_3_) at 7.26 ppm, integration, multiplicities (s = singlet, d = doublet, t = triplet, q = quartet, m = multiplet), and coupling constants (Hz). The ^13^C-NMR spectra are reported in ppm relative to the central line of the triplet for CDCl_3_ at 77.16 ppm. APCI-MS spectra were obtained using a JMS-T100LC instrument (JEOL). Thin-layer chromatography (TLC) analysis of the reaction mixture was performed on Merck silica gel 60 F254 TLC plates and visualized under UV light. Column chromatography on SiO_2_ was performed using Kanto Silica Gel 60 (63–210 μm). UV and visible light irradiations were performed with an LED lamp (PER-AMP, Techno Sigma Co., Ltd. Okayama, Japan). Commercial LED lamps (PER-AMP, Techno Sigma Co., Ltd.) were used as a light source to irradiate the Schlenk tube at a distance of 0.5 cm with aluminum foil covering the outside of the tube. A water bath was used for cooling the setup. A thermo-stainless-steel chamber ensured a constant temperature of 25 °C during the reaction. The temperature inside the chamber was also monitored during the experiment to ensure no fluctuations and kept at 25 °C.

#### 3.2.2. Synthetic Procedure of N-methylnaphthalen-2-amine (**1c**)

Compound **1c** was prepared according to the literature procedure [35]. In a dry 100 mL steel bomb, we added 2-naphthol (500 mg, 3.47 mmol), ammonium chloride (408.5 mg, 7.6 mmol), methylamine (40% in methanol), and ethanol (2.0 M, 1.8 mL). The reaction mixture was heated up to 200 °C, for 12 h. The reaction was quenched by 6 N NaOH after completion. Then, we filtered the residue and extracted it with EtOAc, dried over Na_2_SO_4_, and concentrated under reduced pressure. The residue was purified by silica column chromatography (eluent: n-hexane/EtOAc = 20/1) to afford the desired product *N*-methylnaphthalen-2-amine (**1c**) in 80% yield as yellow oil. **^1^H NMR** (400 MHz, CDCl_3_): *δ* 7.69 (d, *J* = 7.6 Hz, 1H), 7.64 (t, *J* = 8.4 Hz, 2H), 7.40–7.36 (m, 1H), 7.21 (td, *J* = 7.6, 1.2 Hz, 1H), 6.89 (dd, *J* = 8.4, 2.3 Hz, 1H), 6.81 (d, *J* = 2.3 Hz, 1H), 3.88 (s, 1H), 2.95 (s, 3H).

#### 3.2.3. General Protocol for the Photocatalytic Hydroamination of Styrene

An oven-dried Schlenk tube equipped with a magnetic stirring bar was charged with naphthylamines (0.2 mmol) and styrene (0.8 mmol). The tube was evacuated and backfilled with oxygen (three times). Then, 1.6 mL of DCM and 0.4 mL of H_2_O were added by syringe under an O_2_ atmosphere. The solution was stirred at 25 °C with the irradiation of a 365 nm UV LED light. After stirring for 24 h, the organic solvent was removed in vacuo, and the remained water phase was extracted with EtOAc. The organic layer was collected and evaporated under vacuum. The residue was purified by column chromatography on silica gel using *n*-hexane/DCM (20/1) as eluent to obtain the desired products.

##### *N*-(1-Phenylethyl)naphthalen-1-amine (**2a**)

According to the general procedure, a mixture of **1a** (28.6 mg, 0.2 mmol) and styrene (0.09 mL, 0.8 mmol) in 2.0 mL DCM/H_2_O (4:1, *v/v*) was added under oxygen, then stirred for 24 h under 365 nm UV LED light irradiation to afford **2a** as yellow oil in 61% yield. **^1^H NMR** (400 MHz, CDCl_3_): *δ* 7.95–7.92 (m, 1H, Ar-CH), 7.80–7.76 (m, 1H, Ar-CH), 7.50–7.42 (m, 4H, Ar-CH), 7.34–7.30 (m, 2H, Ar-CH), 7.25–7.21 (m, 1H, Ar-CH), 7.19–7.16 (m, 2H, Ar-CH), 6.37 (dt, *J* = 9.3, 4.1 Hz, 1H, Ar-CH), 4.75 (brs, 1H, NH), 4.68 (q, J = 6.7 Hz, 1H, CH), 1.67 (d, *J* = 6.9 Hz, 3H, CH_3_); **^13^C NMR** (100 MHz, CDCl_3_): *δ* 144.9, 142.0, 134.2, 128.7, 128.7, 127.0, 126.5, 125.8, 125.6, 124.7, 123.2, 119.8, 117.2, 106.0, 53.6, 25.3; **HRMS** (APCI): calcd for C_18_H_17_N: *m/z* [M + H]^+^ 248.1434, found 248.1431.

##### *N*-Phenyl-*N*-(1-phenylethyl)naphthalen-2-amine (**2b**)

According to the general procedure, a mixture of **1b** (43.9 mg, 0.2 mmol), styrene (0.09 mL, 0.8 mmol), and Cs_2_CO_3_ (130 mg, 0.4 mmol) in 2.0 mL DCM/H_2_O (4:1, *v/v*) was added under oxygen, then stirred for 24 h under 365 nm UV LED light irradiation to afford **2b** as yellow oil in 50% yield. **^1^H NMR** (400 MHz, CDCl_3_): *δ* 7.71 (d, *J* = 7.8 Hz, 1H, Ar-CH), 7.64 (d, *J* = 9.0 Hz, 1H, Ar-CH), 7.60 (d, *J* = 7.8 Hz, 1H, Ar-CH), 7.42 (d, *J* = 7.3 Hz, 2H, Ar-CH), 7.39–7.35 (m, 1H, Ar-CH), 7.32–7.28 (m, 3H, Ar-CH), 7.25–7.20 (m, 4H, Ar-CH), 7.05–6.97 (m, 2H, Ar-CH), 6.92 (dd, *J* = 9.0, 0.9 Hz, 2H, Ar-CH), 5.44 (q, *J* = 7.0 Hz, 1H, CH), 1.56 (d, *J* = 7.0 Hz, 3H, CH_3_); **^13^C NMR** (100 MHz, CDCl_3_): *δ* 146.9, 144.9, 143.9, 134.5, 129.4, 129.2, 128.7, 128.5, 127.6, 127.2, 127.1, 127.0, 126.2, 124.0, 124.0, 123.9, 122.3, 118.0, 58.1, 19.6; **HRMS** (APCI): calcd for C_24_H_21_N: *m/z* [M + H]^+^ 324.1747, found 324.1752.

##### *N*-Phenyl-1-(1-phenylethyl)naphthalen-2-amine (**2b’**)

According to the general procedure, a mixture of **1b** (43.9 mg, 0.2 mmol), styrene (0.09 mL, 0.8 mmol), and Cs_2_CO_3_ (130 mg, 0.4 mmol) in 2.0 mL DCM/H_2_O (4:1, *v*/*v*) was added under oxygen, then stirred for 24 h under 365 nm UV LED light irradiation to afford **2b’** as yellow oil in 46% yield. **^1^H NMR** (400 MHz, CDCl_3_): *δ* 8.11 (d, *J* = 8.7 Hz, 1H, Ar-CH), 7.82 (d, *J* = 6.9 Hz, 1H, Ar-CH), 7.70 (d, *J* = 8.7 Hz, 1H, Ar-CH), 7.48–7.28 (m, 7H, Ar-CH), 7.22 (d, *J* = 6.9 Hz, 1H, Ar-CH), 7.15 (dd, *J* = 8.5, 7.6 Hz, 2H, Ar-CH), 6.80 (t, *J* = 7.6 Hz, 1H, Ar-CH), 6.64 (d, *J* = 7.6 Hz, 2H, Ar-CH), 5.28 (brs, 1H, NH), 5.24 (t, J = 7.1 Hz, 1H, CH), 1.72 (d, *J* = 7.6 Hz, 3H, CH_3_); **^13^C NMR** (100 MHz, CDCl_3_): *δ*144.5, 144.3, 137.9, 133.2, 131.0, 129.2, 128.8, 127.9, 126.9, 126.5, 126.2, 123.9, 123.3, 119.7, 115.9, 35.6, 16.8; **HRMS** (APCI): calcd for C_24_H_21_N: *m/z* [M + H]^+^ 324.1747, found 324.1752.

##### *N*-Methyl-*N*-(1-phenylethyl)naphthalen-2-amine (**2c**)

According to the general procedure, a mixture of **1c** (31.4 mg, 0.2 mmol) and styrene (0.09 mL, 0.8 mmol) in 2.0 mL DCM/H_2_O (4:1, *v/v*) was added under oxygen, then stirred for 24 h under 365 nm UV LED light irradiation to afford **2c** as yellow oil in 30% yield. **^1^NMR** (400 MHz, CDCl_3_): *δ* 7.71 (t, *J* = 8.5 Hz, 2H, Ar-CH), 7.64 (d, *J* = 8.5 Hz, 1H, Ar-CH), 7.40–7.28 (m, 7H, Ar-CH), 7.23–7.19 (m, 1H, Ar-CH), 7.01 (d, *J* = 2.3 Hz, 1H, Ar-CH), 5.29 (q, *J* = 6.9 Hz, 1H, CH), 2.76 (s, 3H, CH_3_), 1.60 (d, *J* = 6.9 Hz, 3H, CH_3_); **^13^C NMR** (100 MHz, CDCl_3_): *δ* 144.7, 144.6, 133.2, 129.0, 128.8, 128.4, 128.0, 126.9, 126.6, 126.3, 122.0, 121.6, 121.0, 115.0, 35.1, 31.5, 15.7; **HRMS** (APCI): calcd for C_19_H_19_N: *m/z* [M + H]^+^ 262.1590, found 262.1591.

##### *N*-Methyl-*N*-(1-phenylethyl)naphthalen-2-amine (**2c’**)

According to the general procedure, a mixture of **1c** (31.4 mg, 0.2 mmol) and styrene (0.09 mL, 0.8 mmol) in 2.0 mL DCM/H_2_O (4:1, *v/v*) was added under oxygen, then stirred for 24 h under 365 nm UV LED light irradiation to afford **2c’** as yellow oil in 22% yield. **^1^H NMR** (400 MHz, CDCl_3_): *δ* 7.97 (d, *J* = 8.2 Hz, 1H, Ar-CH), 7.75 (dd, *J* = 12.8, 8.0 Hz, 2H, Ar-CH), 7.40 (t, *J* = 8.0 Hz, 1H, Ar-CH), 7.30 (d, *J* = 4.6 Hz, 4H, Ar-CH), 7.25–7.10 (m, 3H, Ar-CH), 5.16 (q, *J* = 7.3 Hz, 1H, NH), 2.70 (s, 3H, CH_3_), 1.77 (d, *J* = 7.3 Hz, 3H, CH_3_); **^13^C NMR** (100 MHz, CDCl_3_): *δ* 148.3, 142.6, 135.2, 128.9, 128.5, 127.5, 127.1, 126.4, 126.3, 122.2, 117.1, 107.3, 57.1, 32.2, 16.4; **HRMS** (APCI): calcd for C_19_H_19_N: *m/z* [M + H]^+^ 262.1590, found 262.1591.

#### 3.2.4. Procedure for “ON-OFF” Experiment

The “ON-OFF” experiment was conducted by employing **1a** (28.6 mg, 0.2 mmol) and styrene (0.09 mL, 0.8 mmol) in DCM/H_2_O (2.0 mL, 4/1) under 365 nm UV irradiation in an oxygen atmosphere. Under the UV light irradiation to the mixture, the reaction proceeded to form the corresponding product **2a**. However, no light irradiation led to suppressing the reaction completely. Finally, continuous 365 nm UV light resulted in consuming **1a** to give **2a** with 61% yield.

#### 3.2.5. Procedures for UV–Vis Absorbance Analysis

**1a** and styrene were employed in the binary solvent system. The color of the reaction mixture immediately changed from colorless to an orange color, and finally to brown in 5 min at the irradiation of 365 nm UV LED under O_2_ atmosphere (see Supporting Information). In order to understand the generation of EDA complex between substrate **1a** and oxygen, a series of UV–vis absorption measurements were carried out. Initially, we took the optical absorption spectra of **1a** and styrene in DCM (10 μM) and then measured the mixture of **1a** and styrene (1:1 *v/v*) in DCM (10 μM). The UV–vis absorption spectra indicated that no electron donor–acceptor (EDA) complex formed between the two starting materials. Next, we checked the UV–vis absorption of **1a** and oxygen. The sample was prepared by oxygen bubbling for 10 min in the DCM solution of **1a** (10 μM). An obvious increasing bathochromic displacement and absorption can be observed in the spectrum, which supported the formation of the EDA complex between **1a** and O_2_.

## 4. Conclusions

In summary, we have developed a new approach for the Markovnikov-selective hydroamination of styrene with naphthylamines by an irradiation of UV LED light (365 nm) via an in situ electron donor–acceptor (EDA) complexation between naphthylamines and oxygen. This protocol is eco-friendly, green, and facile, showcasing synthetic potential for aerobic C–N bond formation without using any metal catalyst or photosensitizer. The exploration of more C–N bond formation reactions involving naphthylamines and other alkenes is ongoing in our group.

## Figures and Tables

**Table 1:** Optimization of reaction conditions.

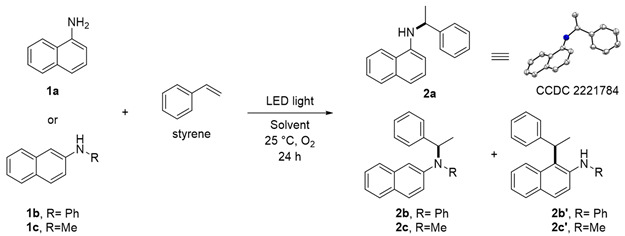						
Entry	Substrate	Molar Ratio of Substrate (1/styrene)	Wavelength Λ (nm)	Solvent	Additives	Yield ^a^ (%)
1	**1a**	1/1	365	DCM ^b^/H_2_O = 4/1	-	5
2	**1a**	1/2	365	DCM/H_2_O = 4/1	-	24
3	**1a**	1/8	365	DCM/H_2_O = 4/1	-	10
4	**1a**	1/4	340	DCM/H_2_O = 4/1	-	**2a**: 11
5	**1a**	1/4	365	DCM/H_2_O = 4/1	-	**2a**: 61 [61 ^c^]
6	**1a**	1/4	448	DCM/H_2_O = 4/1	-	*N.R.* ^d^
7	**1a**	1/4	365	MeCN/H_2_O = 4/1		**2a**: 2
8	**1a**	1/4	365	Acetone/H_2_O = 4/1	-	**2a**: 13
9	**1a**	1/4	365	EtOAc/H_2_O = 4/1	-	**2a**: 13
10	**1b**	1/4	365	H_2_O	-	**2b**: 8; **2b’**: 26
11	**1b**	1/4	365	DCM	-	**2b**: 15; **2b’**: 23
12	**1b**	1/1	365	DCM/H_2_O = 4/1	-	**2b**: 7; **2b**’: 7
13	**1b**	1/2	365	DCM/H_2_O = 4/1	-	**2b**:15, **2b’**: 15
14	**1b**	1/4	365	DCM/H_2_O = 4/1	-	**2b**: 39, **2b’**: 51
15	**1b**	1/4	365	DCM/H_2_O = 4/1	Cs_2_CO_3_ (2.0 equiv.)	**2b**: 50, **2b’**: 46
16	**1b**	1/4	365	DCM/H_2_O = 4/1	DBU (2.0 equiv.)	**2b**: 25, **2b’**: 30
17	**1b**	1/4	365	DCM/H_2_O = 4/1	Et_3_N (2.0 equiv.)	**2b**: 25, **2b’**: 27
18	**1b**	1/4	460	DCM/H_2_O = 4/1	9-fluorene (2.0 equiv.)	*N.R.*
19	**1b**	1/4	460	DCM/H_2_O = 4/1	rose bengal (2.0 equiv.)	
20	**1b**	1/4	460	DCM/H_2_O = 4/1	4CzIPN (2.0 equiv.)	
21	**1b**	1/4	460	DCM/H_2_O = 4/1	eosin Y (2.0 equiv.)	
22	**1c**	1/4	365	DCM/H_2_O = 4/1	-	**2c**: 30, **2c’**: 22
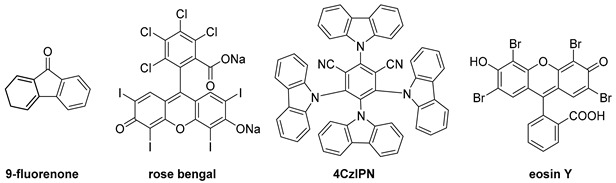						

^a^ NMR yield using 1,3,5-trimethoxybenzene as the internal standard. ^b^ DCM = dichloromethane. ^c^ isolated yield. ^d^ *N.R.* = no reaction.

## Data Availability

CIF of the crystal of **2a** is available as Supplementary. The X-ray crystallographic coordinate for the structure reported in this study has been deposited at the Cambridge Crystallographic Data Centre (CCDC) under deposition numbers CCDC- 2221784 (**2a**). These data can be obtained free of charge from The Cambridge Crystallographic Data Centre via www.ccdc.cam.ac.uk/data_request/cif (accessed on 24 December 2022).

## Supplementary Materials

The following supporting information can be downloaded at: https://www.mdpi.com/article/10.3390/molecules28010356/s1, Figure S1: Reaction setup and LED apparatus; Figure S2: “On-off” experiment; Figure S3: Visual characterization of the reaction mixture under UV irradiation; Figure S4: UV-Vis absorption analysis; Figure S5: Proposed mechanism of photoinduced C-N bond formation of 1**b** and styrene; Figure S6: Compound **2a** (^1^H NMR, 400 MHz, CDCl_3_); Figure S7: Compound **2a** (^13^C NMR, 100 MHz, CDCl_3_): Figure S8: Compound **2b** (^1^H NMR, 400 MHz, CDCl_3_); Figure S9: Compound **2b** (^13^C NMR, 100 MHz, CDCl_3_); Figure S10: Compound **2b’** (^1^H NMR, 400 MHz, CDCl_3_); Figure S11: Compound **2b’** (^13^C NMR, 100 MHz, CDCl_3_); Figure S12: Compound **2c** (^1^H NMR, 400 MHz, CDCl_3_); Figure S13: Compound **2c** (^13^C NMR, 100 MHz, CDCl_3_); Figure S14: Compound **2c’** (^1^H NMR, 400 MHz, CDCl_3_); Figure S15: Compound **2c’** (^13^C NMR, 100 MHz, CDCl_3_); Figure S16: Single crystal structure of CCDC 2221784 (**2a**); Table S1: Optimization of reaction conditions; Table S2: On-off experiment; Table S3: Crystal information of CCDC 2221784 (**2a**).
