# Supplementary material for: Metal-Free Aerobic C–N Bond Formation of Styrene and Arylamines via Photoactivated Electron Donor–Acceptor Complexation
Source: Molecules. 2023 Jan 1;28(1):356. doi: 10.3390/molecules28010356 (PMC9822123; doi:10.3390/molecules28010356)
Supplement: Supplementary file 1 [file molecules-28-00356-s001.zip › molecules-2099899-supplementary.pdf]

Supplementary Material

Metal-Free Aerobic C–N Bond Formation of Styrene and Arylamines via Photoactivated Electron Donor–Acceptor Complexation

Duona Fan, Ahmed Sabri, Hiroaki Sasai and Shinobu Takizawa *

The Institute of Scientific and Industrial Research (SANKEN), Osaka University, Mihogaoka, Ibaraki-shi 567-0047, Osaka, Japan

* Correspondence: taki@sanken.osaka-u.ac.jp; Tel.: +81-6-6879-8467

1. General information

^{1}H-, and ^{13}C-NMR were recorded with JEOL JMN ECS400 FT NMR, or Bruker AVANCE II (^{1}H-NMR 400 MHz, ^{13}C-NMR 100 MHz) ^{1}H-NMR spectra are reported as follows: a chemical shift in ppm downfield of tetramethylsilane (TMS) and referenced to residual solvent peak ($CDCl_3$) at 7.26 ppm, integration, multiplicities (s = singlet, d = doublet, t = triplet, q = quartet, m = multiplet), and coupling constants (Hz). ^{13}C-NMR spectra reported in ppm relative were to the central line of triplet for $CDCl_3$ at 77.16 ppm, ESI-MS spectra were obtained with JMS-T100LC (JEOL). Thin-layer chromatography (TLC) analysis of reaction on mixtures was performed using Merck silica gel 60 F254 TLC plates and visualized under UV. Column chromatography on SiO_2 was performed with Kanto Silica Gel 60 (63-210 μm). UV and visible light irradiations were performed with LED lamp (PER-AMP, Techno Sigma Co., Ltd.).

1-Naphthylamine (**1a**), *N*-Phenyl-2-napthylamine (**1b**) and styrene was purchased from Tokyo Chemical Industries (TCI). All commercially available organic and inorganic compounds were used directly without further purification.

Commercial LED lamps (PER-AMP, Techno Sigma Co., Ltd.) are used as light source to irradiate the Schlenk tube at a distance of 0.5 cm with aluminum foil covering outside of the tube (Figure S1). A water bath is used for cooling the setup. A thermo-stainless-steel chamber ensures constant at 25 °C during the reaction. Temperature inside the chamber was also monitored during the experiment to ensure no fluctuations and kept at 25 °C.

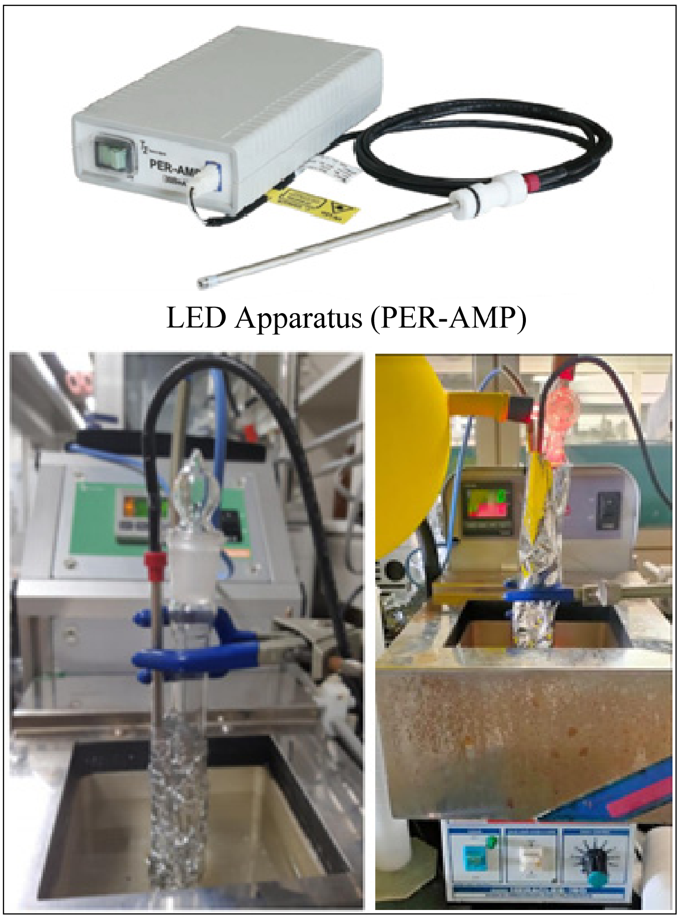

Reaction setup using UC reactor and LED

Figure S1. Reaction setup and LED apparatus.

2. Optimization of reaction conditions

N-Phenyl-2-napthylamine (**1b**) with styrene were employed as model substrate to screen the reaction conditions (Table S1).

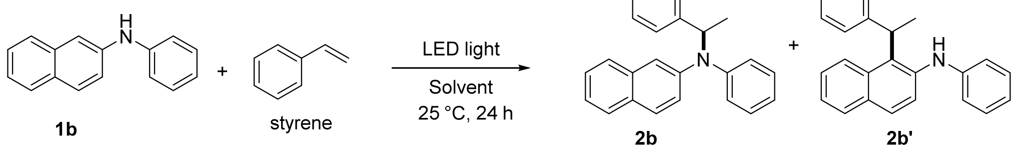

Table S1. Optimization of reaction conditions.

Entry	Wavelength λ(nm)	Molar ratio of substrate (1b/styrene)	Solvent	Additives	Yield[a] (%) 2b	2b′
1	-	1:1	DCM	-	*N.R.*[b]	
2	365	1:1	DCM	-	8	6
3	280	1:1	DCM	-	Trace	
4	340	1:1	DCM	-	Trace	
5	385	1:1	DCM	-	1	2
6	395	1:1	DCM	-	Trace	
7	448	1:1	DCM	-	*N.D.*[c]	
8	365	1:3	DCM	-	12	10
9	365	1:4	DCM	-	15	23
10	365	1:5	DCM	-	10	11
11	365	1:8	DCM	-	5	9
12	365	1:4	DCM	TFA (1.0 M)	Trace	
13	365	1:4	DCM/HFIP (3/1)	-	11	20
14	365	1:4	DCM/H_2O (1/2)	-	29	37
15	365	1:4	DCM/H_2O (2/1)	-	38	47
16	365	1:4	DCM/H_2O (4/1)	-	39	51
17[d]	365	1:4	DCM/H_2O (4/1)	-	1	3
18[e]	365	1:4	DCM/H_2O (4/1)	-	2	4
19	365	1:4	DCM/MeOH (4/1)	-	15	13
20	365	1:4	DCM/EtOH (4/1)	-	11	6
21	365	1:4	H_2O	-	8	26
22	365	1:4	Toluene	-	9	14
23	365	1:4	MeCN/H_2O (4/1)	-	11	8
24	365	1:4	DCE/H_2O (4/1)	-	12	16
25	365	1:4	Acetone/H_2O (4/1)	-	3	5
26	365	1:4	1,4-dioxane/H_2O (4/1)	-	6	5
27	365	1:4	DCM/H_2O (4/1)	$Na_2S_2O_3$ (0.5 eq.)	17	14
28	365	1:4	DCM/H_2O (4/1)	Bu_4NClO_4 (0.1 M)	39	51
29	365	1:4	DCM/H_2O (4/1)	Bu_4NPF_6 (0.1 M)	10	13
30	365	1:4	DCM/H_2O (4/1)	Et_3N (2 eq.)	25	27
31	365	1:4	DCM/H_2O (4/1)	DMSO (0.1 M)	19	20
32	365	1:4	DCM/H_2O (4/1)	$NaHCO_3$ (1.0 eq.)	23	21
33	365	1:4	DCM/H_2O (4/1)	Cs_2CO_3 (2.0 eq.)	50	46
34	365	1:4	DCM/H_2O (4/1)	TMSCl (1.0 eq.)	38	40
35	365	1:4	DCM/H_2O (4/1)	$(CH_3O)_2CO$ (2.0 eq.)	19	16

[a] NMR yield; [b] N.R.= no reaction; [c] N.D.= not detected; [d] Under N_2 conditions; [e] Under air conditions.

3. Mechanism study

3.1. On-off experiment

ON-OFF experiment was conducted by employing **1a** (28.6 mg, 0.2 mmol) and styrene (0.09 mL, 0.8 mmol) in DCM/H_2O (2.0 mL, 4/1) under 365 nm UV irradiation in oxygen atmosphere. Under the UV light irradiation to the mixture, the reaction proceeded to form the corresponding product **2a**. However no light irradiation led to suppressing the reaction completely (Figure S2). Finally, continuous 365nm UV light resulted in consuming **1a** to give **2a** with 61% yield. Table 2 below showed the yield of **2a** from 0 to 24 hours.

Table S2. On-off experiment.

Time(h)	0	1(On)	2(Off)	3(On)	4(Off)	5(On)	6(Off)	7(On)	8(Off)	24(On)
Yield **2a**(%)	0	6	6	8	8	11	11	15	15	61

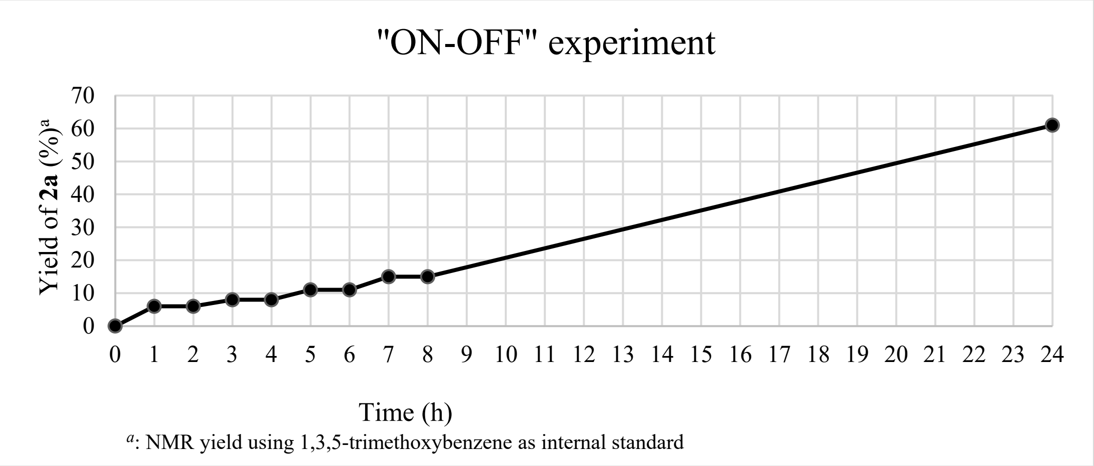

Figure S2. "On-off" experiment.

4.2. UV-vis absorbance spectrum

1a and styrene were employed in the binary solvent system. The color of the reaction mixture immediately changed from colorless to orange color, finally to brown in 5 min at the irradiation of 365 nm UV LED under O_2 atmosphere (Figure S3).

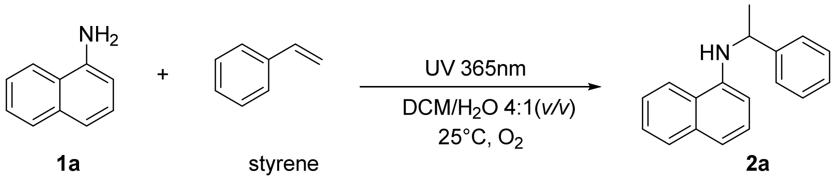

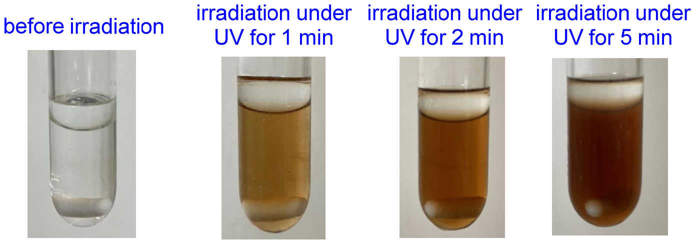

Figure S3. Visual characterization of the reaction mixture under UV irradiation.

In order to understand the generation of EDA complex between substrate **1a** and oxygen, series of UV-vis absorption measurement was carried out. Initially, we took the optical absorption spectra of **1a** and styrene in DCM (10 μM) and then measured the mixture of **1a** and styrene (1:1 *v*/*v*) in DCM (10 μM). The UV-vis absorption spectra indicated

that no electron donor-receptor (EDA) complex formed between two starting materials. Next, we checked the UV-vis absorption of **1a** and oxygen. The sample was prepared by oxygen bubbling 10 min in the DCM solution of **1a** (10 μM). An obvious increasing of bathochromic displacement and absorption can be observed in spectrum, which supported the formation of EDA complex between **1a** and O_2 (Figure S4).

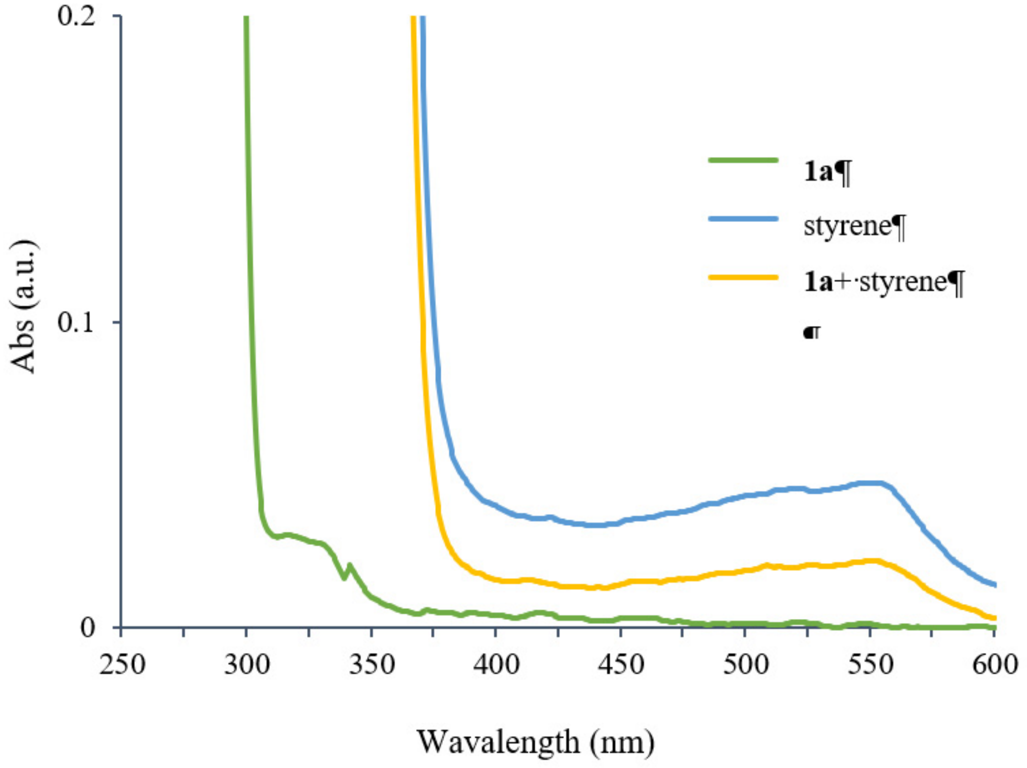

(a) UV–vis absorbance spectra of **1a**, styrene, and mixture of **1a** and styrene in DCM

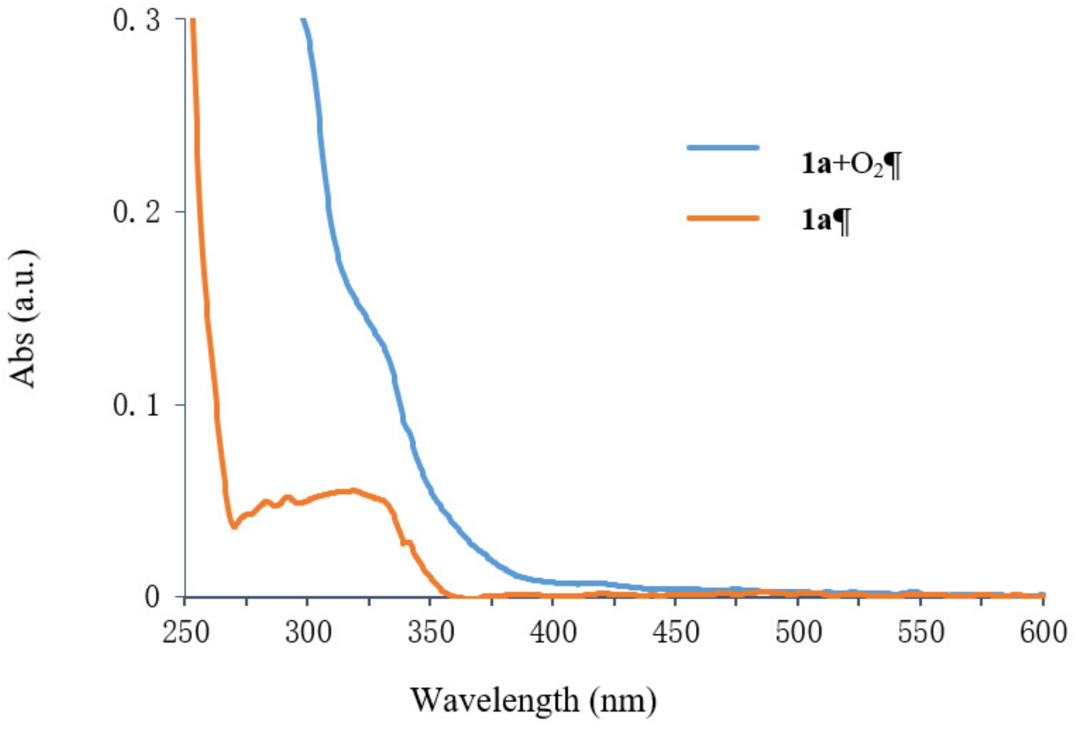

(b) UV–vis absorbance spectra of **1a** and mixture of **1a** and O_2 in DCM.

Figure S4. UV-Vis absorption analysis.

On the basis of the preliminary mechanism studies, a plausible mechanism is herein presented in **Figure S5** concerning the formation of naphthylamine radical. There are two possible pathways according to our hypothesis. When applying *N*-phenyl-2-naphthylamine **1b** as substrate, **Path a** depicted a direct generation of naphthylamine radical promoting by 365 nm UV LED light, due to its obvious UV absorption in the range of 300-400 nm. Mechanism of **Path b** begins with the in situ formation of EDA complex **I**, triggering by UV light, the formation of naphthylamine radical is improved. The generated **IIA** and **IIB** species are in equilibrium by electron transfer, after the bond-formation of styrene with **IIA** and **IIB** led to the corresponding C–N formation product **2b** and C–C formation product **2b'**, respectively. For our hypothesis, Path a and path b may both contribute to the photochemical reaction. When **1a** was used as the substrate, only C–N formation occurred, Path c showed the proposed mechanism for C-N bond formation of **1a** and styrene, owing to the sole generation of N cation radicals **VI**, only C-N bond formation product **2a** formed.

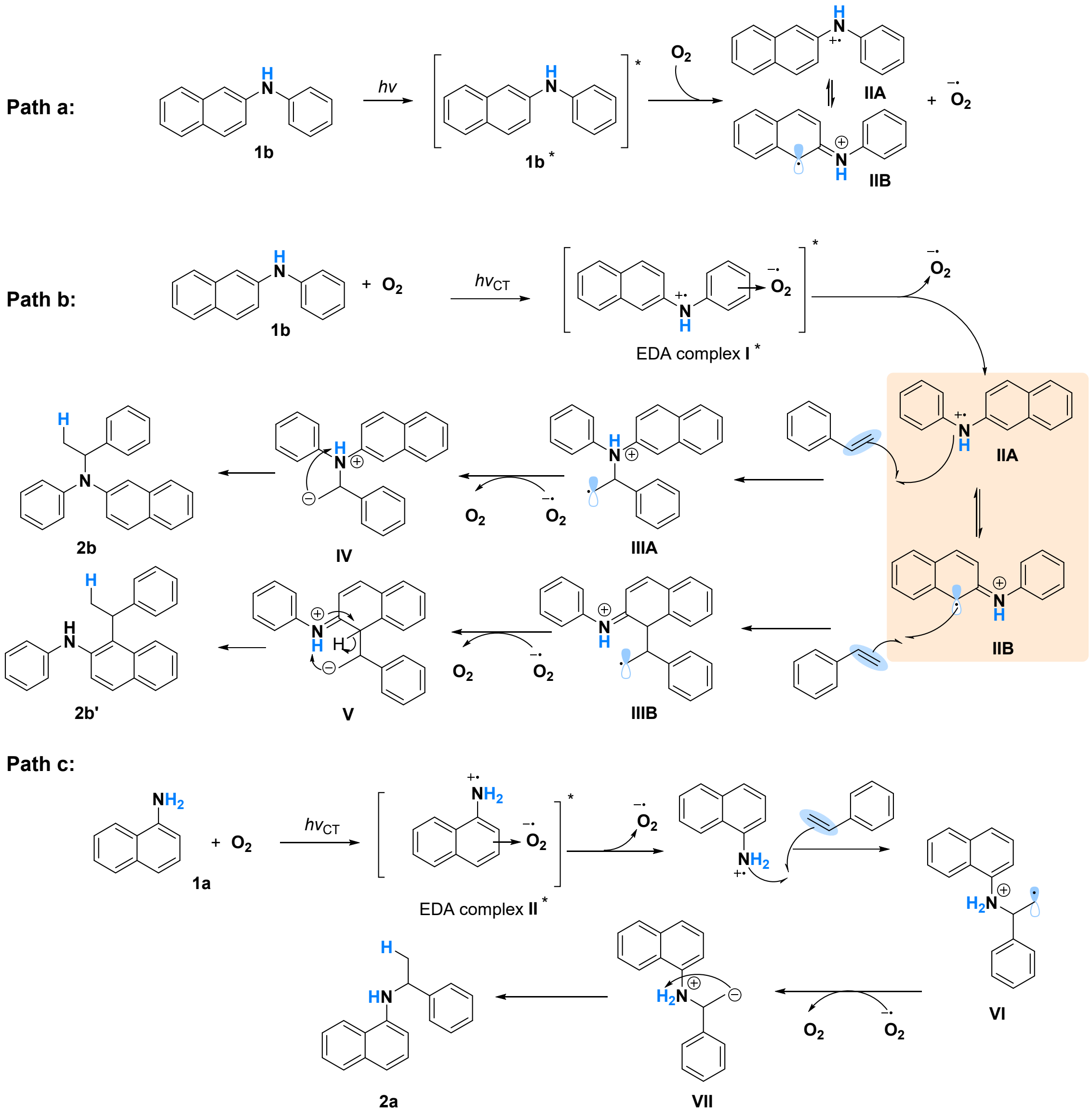

Figure S5. Proposed mechanism of photoinduced C-N bond formation of **1b** and styrene.

4. NMR Spectra

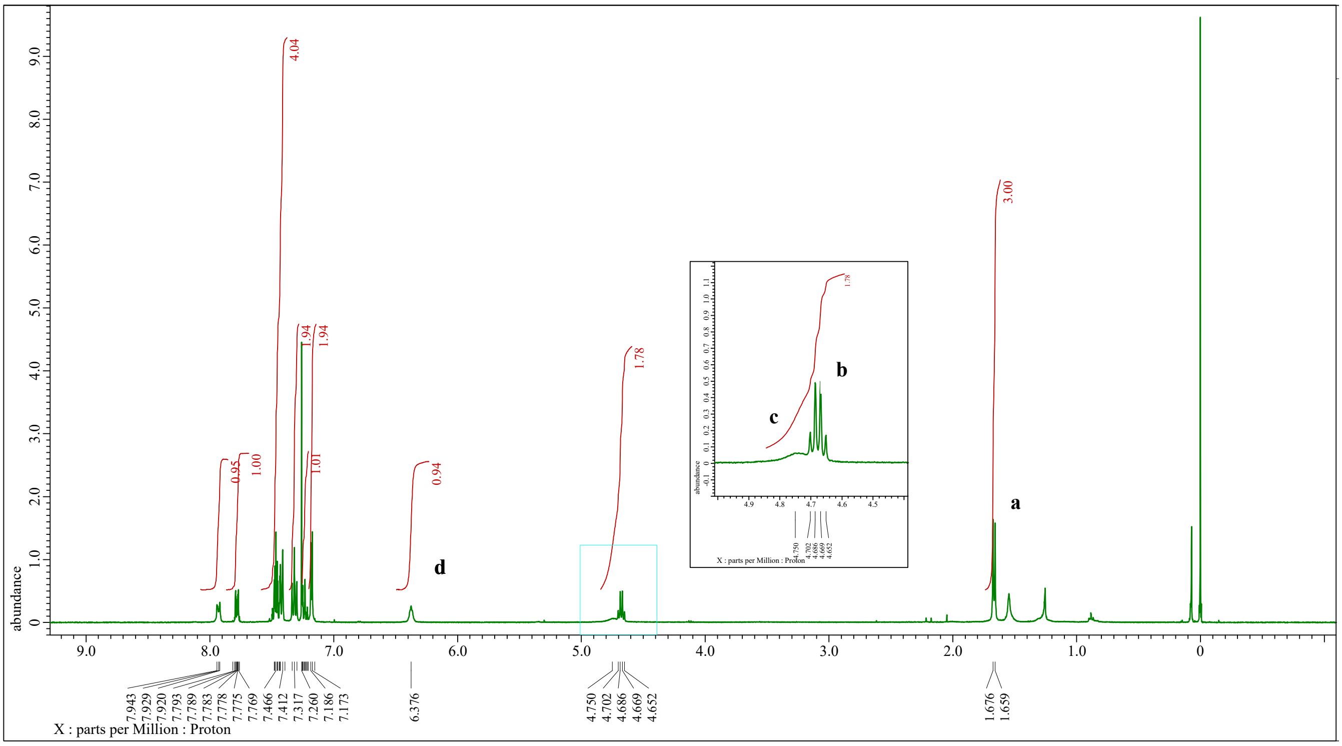

Figure S6. Compound 2a (^{1}H NMR, 400 MHz, $CDCl_3$).

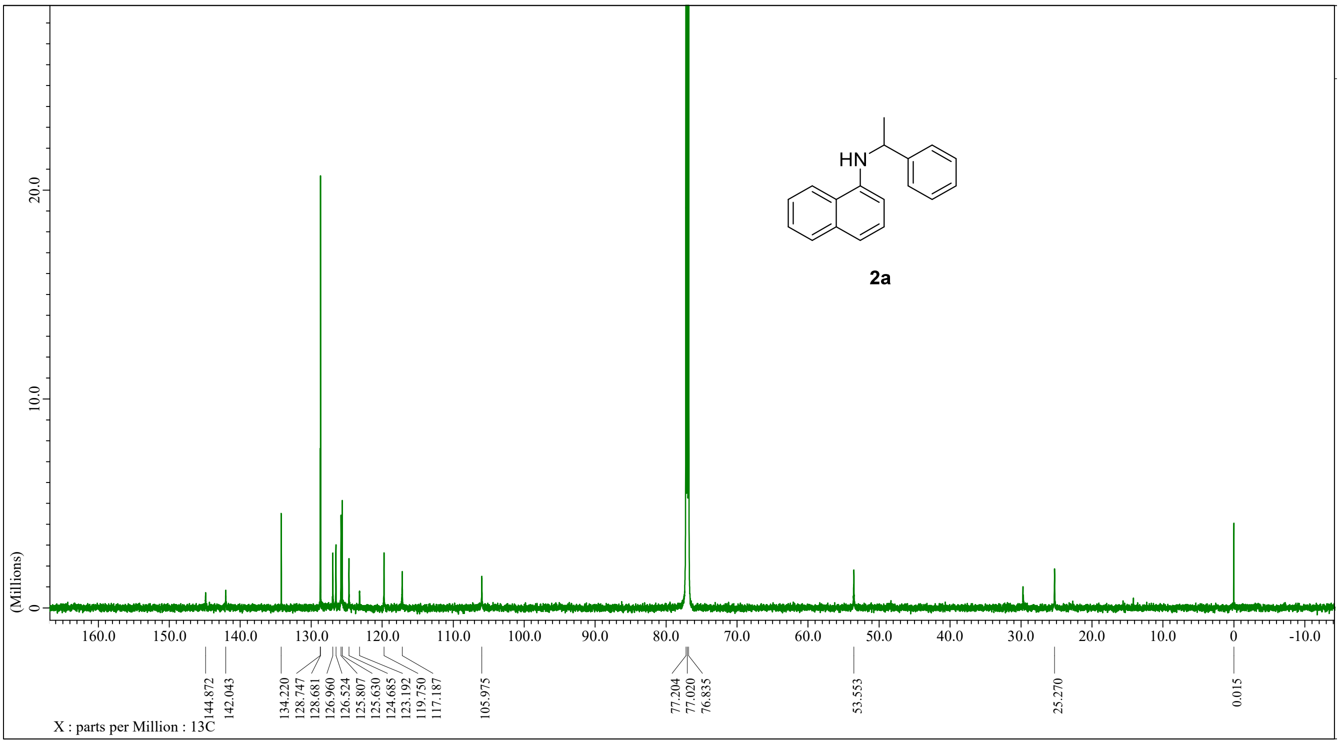

Figure S7. Compound **2a** (^{13}C NMR, 100 MHz, $CDCl_3$).

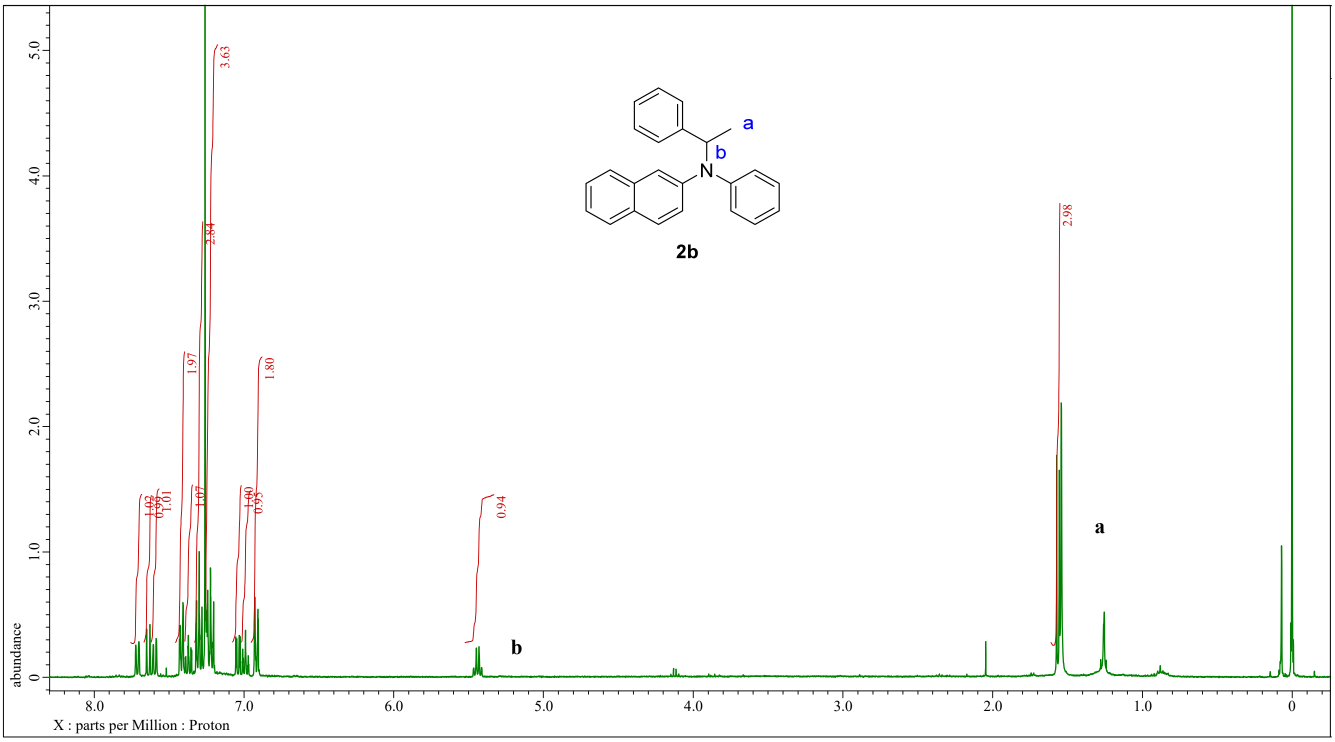

Figure S8. Compound **2b** (^{1}H NMR, 400 MHz, $CDCl_3$).

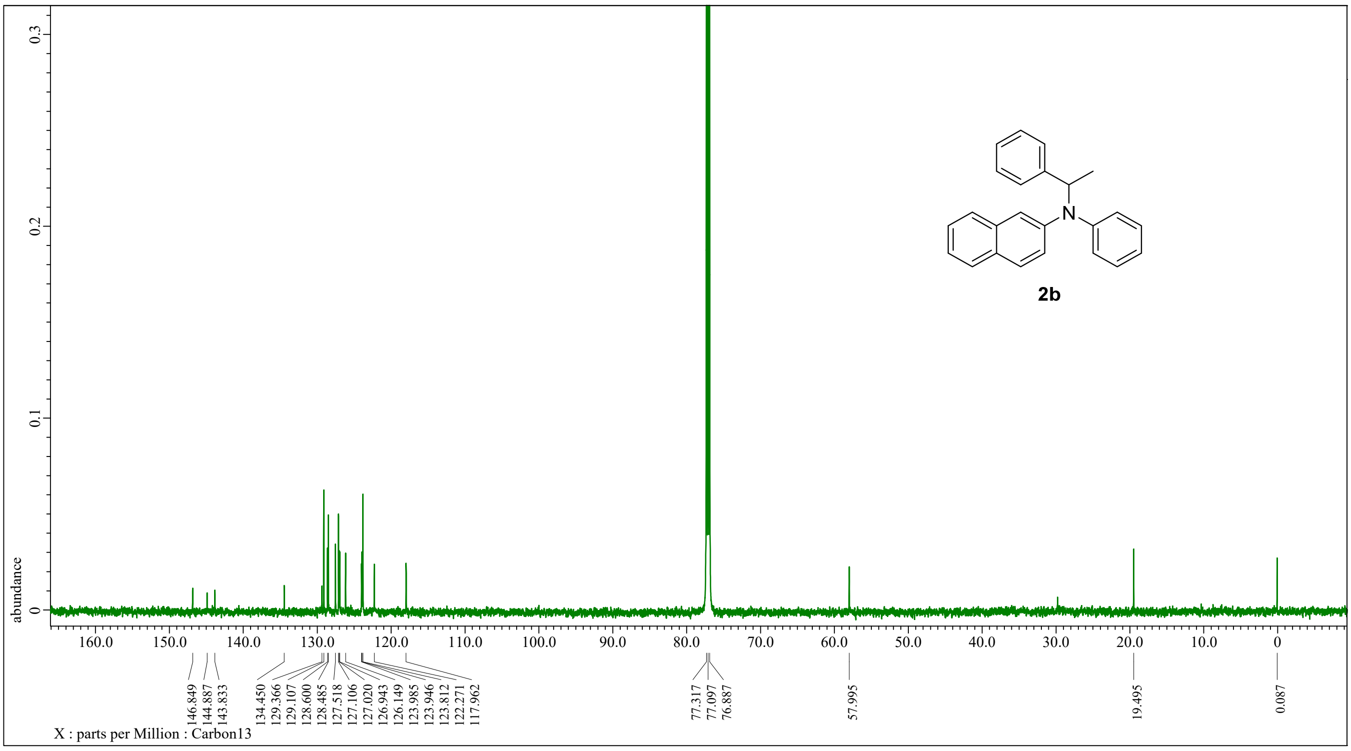

Figure S9. Compound **2b** (^{13}C NMR, 100 MHz, $CDCl_3$).

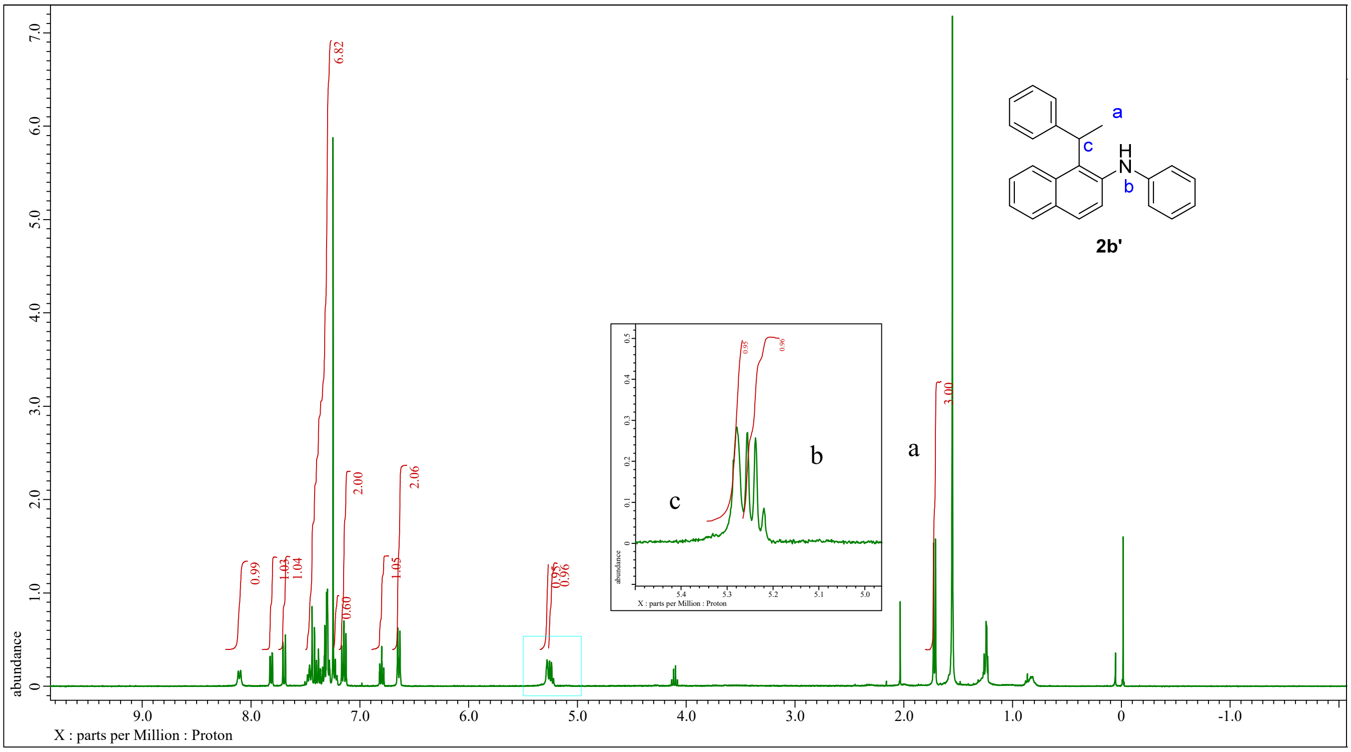

Figure S10. Compound **2b'** (^{1}H NMR, 400 MHz, $CDCl_3$).

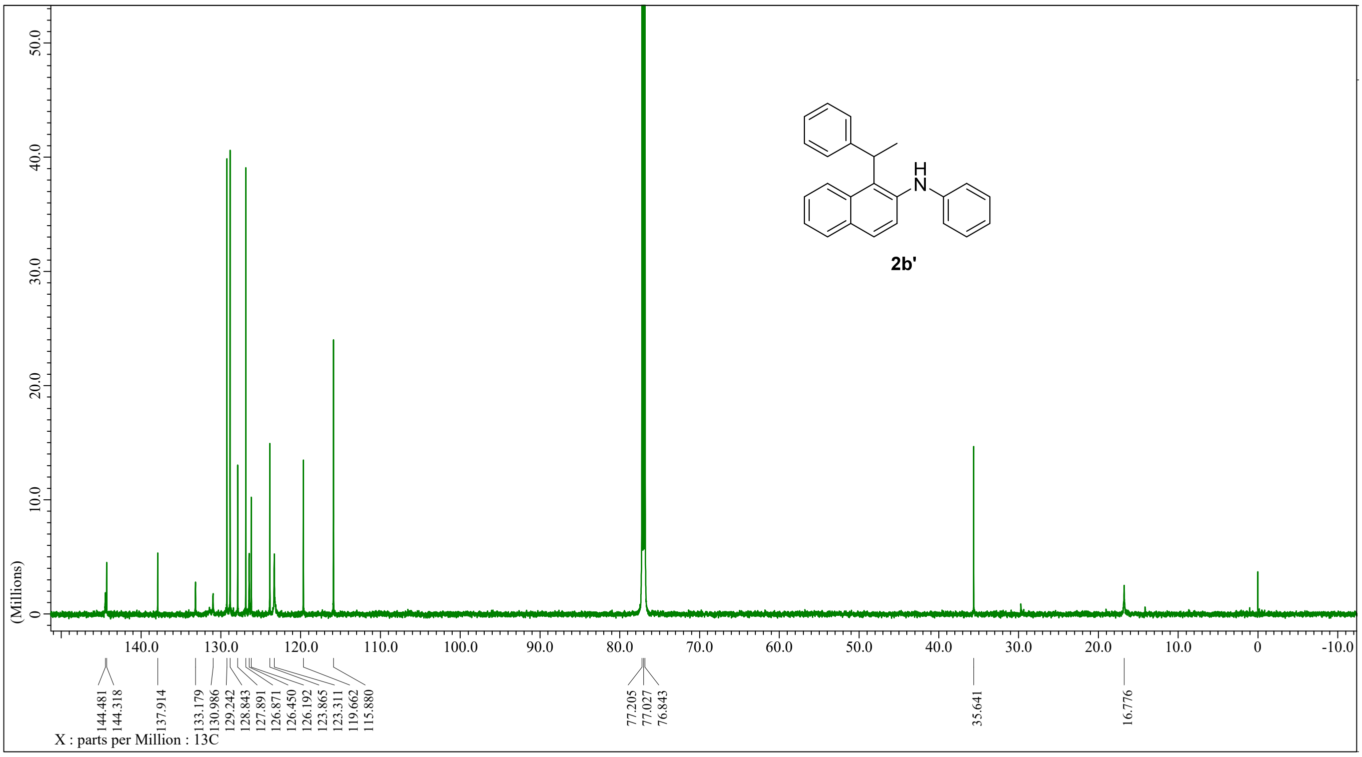

Figure S11. Compound **2b'** (^{13}C NMR, 100 MHz, $CDCl_3$).

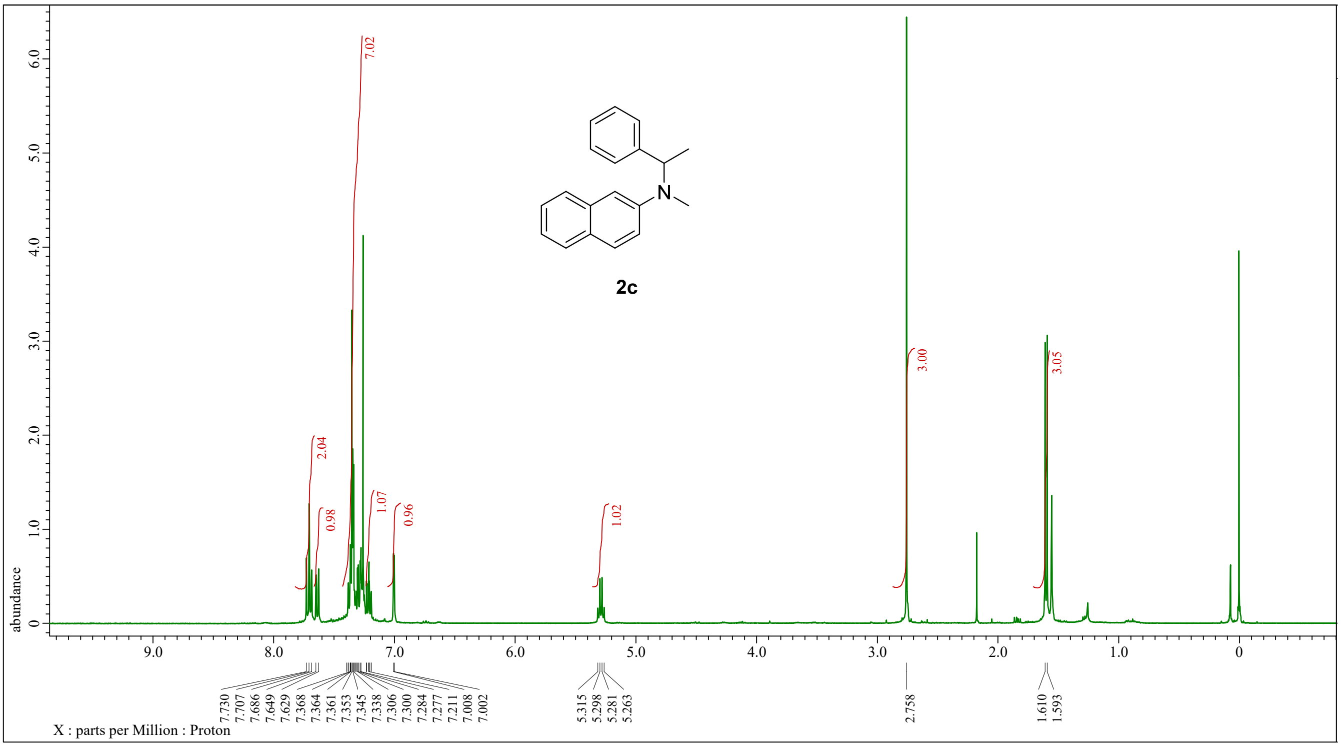

Figure S12. Compound **2c** (^{1}H NMR, 400 MHz, $CDCl_3$).

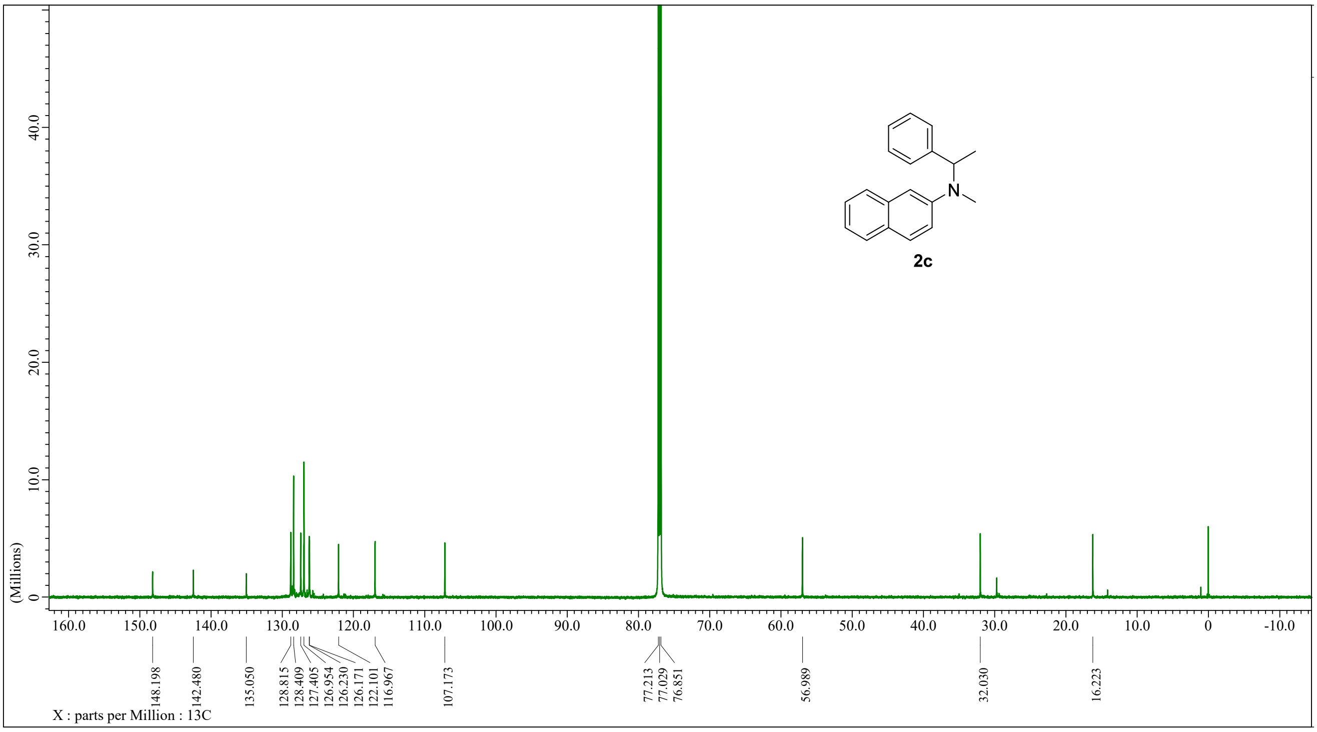

Figure S13. Compound **2c** (^{13}C NMR, 100 MHz, $CDCl_3$).

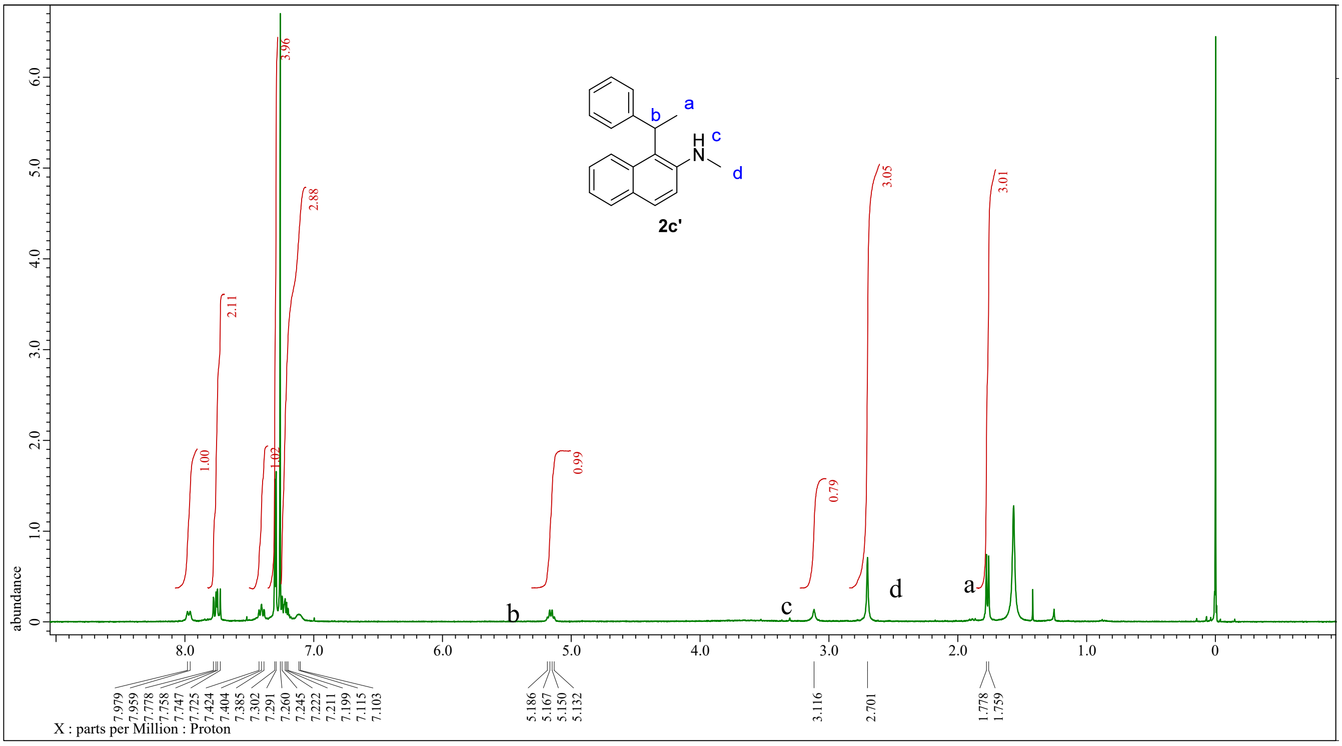

Figure S14. Compound **2c′** (^{1}H NMR, 400 MHz, $CDCl_3$).

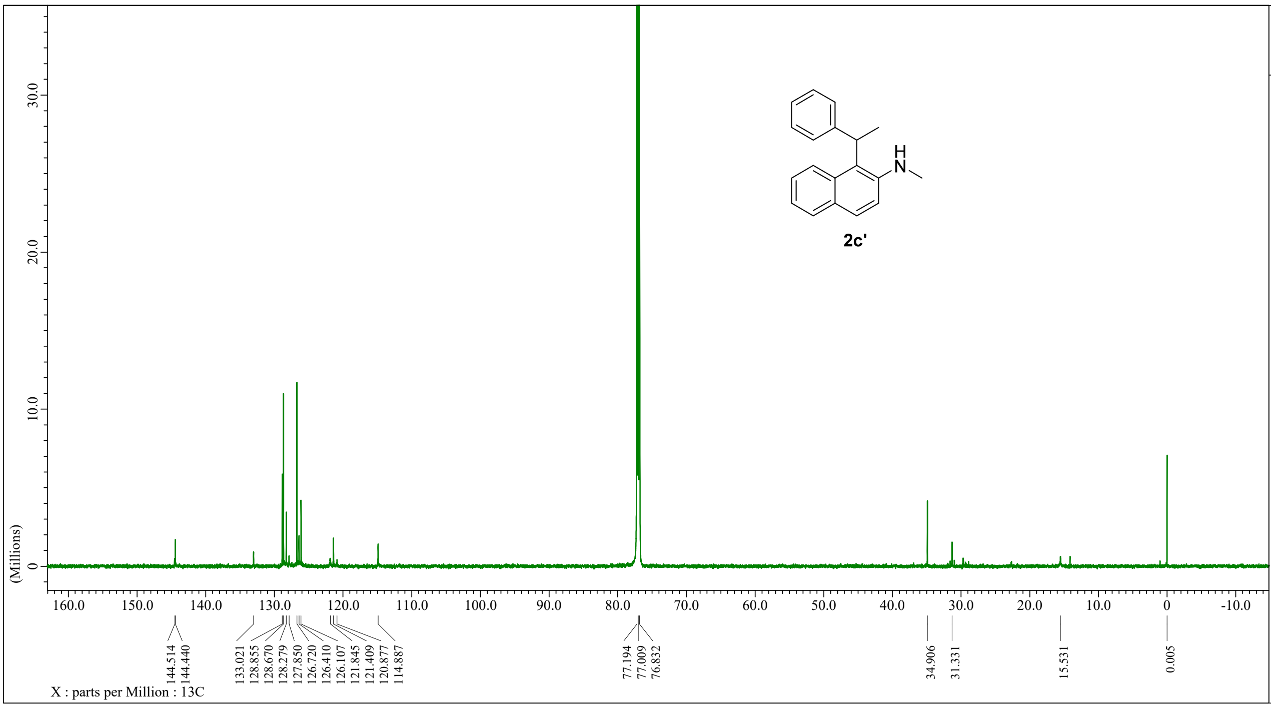

Figure S15. Compound **2c′** (^{13}C NMR, 100 MHz, $CDCl_3$).

7. Crystal information of 2a

Table S3. Crystal information of CCDC 2221784 (**2a**)

**Empirical formula**	$C_{18}H_{17}N$
Formula weight	247.32
Temperature/K	293(2)
Crystal system	Monoclinic
Space group	$P2_1/n$
a/Å	12.0266(2)

b/Å	7.89240(10)
c/Å	14.3664(2)
α/°	90
β/°	94.8760(10)
γ/°	90
Volume/Å^3	1358.71(3)
Z	4
ρ_{calc}g/cm^3	1.209
μ/mm^{-1}	0.531
F(000)	528.0
Crystal size/mm^3	0.139 × 0.061 × 0.023
Radiation	CuKα (λ = 1.54184)
2Θ range for data collection/°	9.214 to 151.226
Index ranges	-13 ≤ h ≤ 15, -9 ≤ k ≤ 9, -18 ≤ l ≤ 17
Reflections collected	17309
Independent reflections	2682 [R_{int} = 0.0469, R_{sigma} = 0.0314]
Data/restraints/parameters	2682/0/173
Goodness-of-fit on F^2	1.091
Final R indexes [I>=2σ (I)]	R_1 = 0.0437, wR_2 = 0.1300
Final R indexes [all data]	R_1 = 0.0556, wR_2 = 0.1380
Largest diff. peak/hole / e Å^{-3}	0.29/-0.23

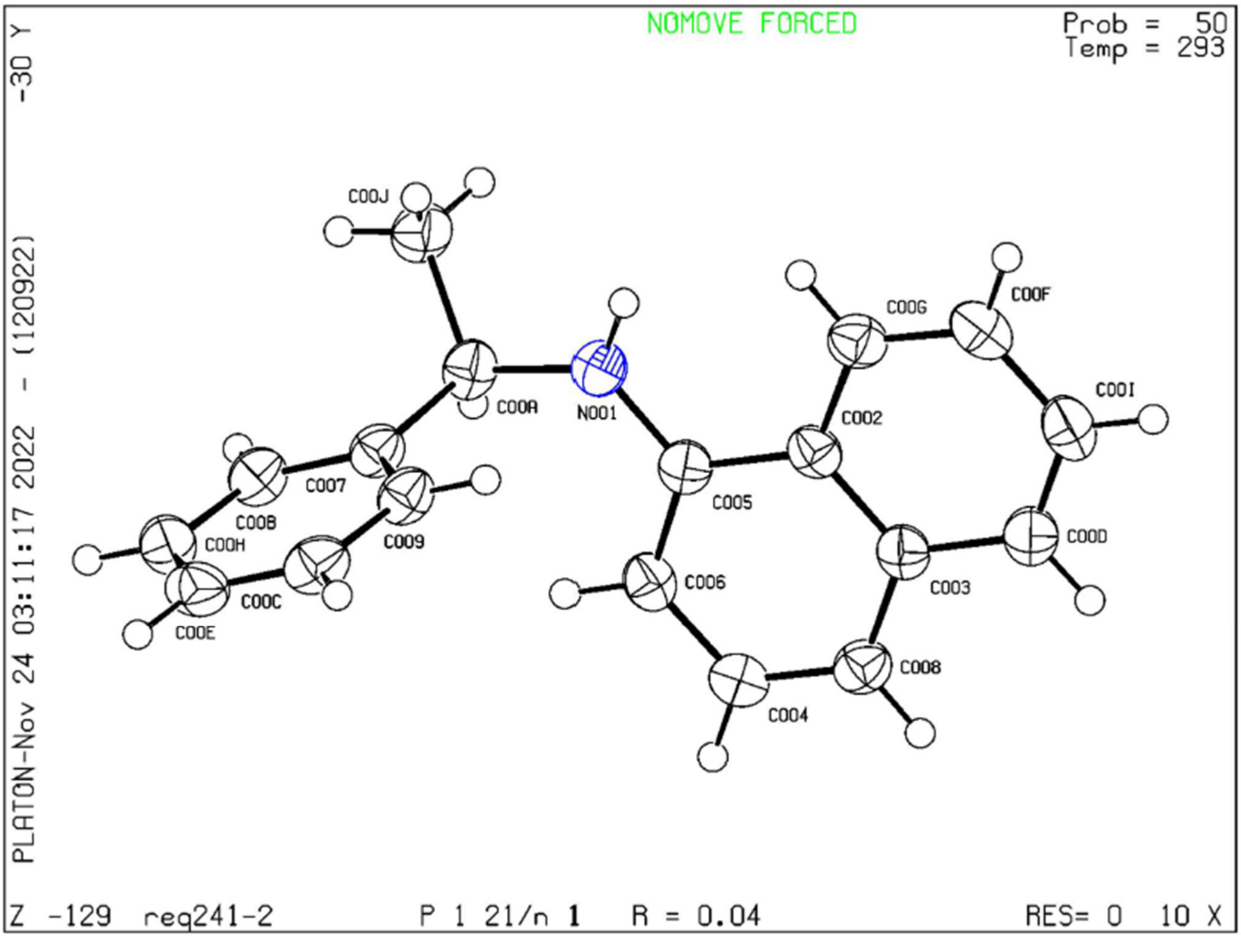

Figure S16. Single crystal structure of CCDC 2221784 (**2a**).
